# From Small Molecules to Polymers: Developing Non-Fullerene Acceptors for Efficient NIR Photothermal Cancer Therapy

**DOI:** 10.3390/polym17243304

**Published:** 2025-12-13

**Authors:** Yulia A. Isaeva, Elizaveta D. Blagodarnaia, Anastasia A. Vetyugova, Maxim E. Stepanov, Liya A. Poletavkina, Ivan V. Dyadishchev, Askold A. Trul, Tatyana V. Egorova, Roman A. Akasov, Yuriy N. Luponosov

**Affiliations:** 1 Enikolopov Institute of Synthetic Polymeric Materials, Russian Academy of Sciences, Profsoyuznaya St. 70, Moscow 117393, Russia; 2 Institute of Physics, Technology, and Informational Systems, Moscow Pedagogical State University, Malaya Pirogovskaya St. 29/7, Building 1, Moscow 119991, Russia; 3 Department of Biochemistry, Petrovsky Medical University, Moscow 119435, Russia

**Keywords:** conjugated polymers, non-fullerene acceptors, organic semiconductor nanoparticles, phototherapy

## Abstract

Developing organic photothermal agents that are highly stable and have tunable electronic properties is important for advancing low-invasive cancer therapy. In this study, we present the synthesis and evaluation of three conjugated photothermal agents inspired by non-fullerene Y-series acceptors: the small molecule BTPT-OD, as well as two of its polymer derivatives with regular (*r*-BTPT) and irregular (*ir*-BTPT) structures. All of the compounds absorb light effectively in the red and near-infrared spectral ranges, with absorption maxima from 734 to 746 nm, and form stable nanoparticles (NPs) via nanoprecipitation, ranging in size from 13 to 39 nm. NPs exhibited negative surface charges, with *ζ*-potentials of −12.9, −15.5, and −17.9 mV for BTPT-OD, *r*-BTPT, and *ir*-BTPT NPs, respectively. Irradiation at a wavelength of 730 nm revealed that *r*-BTPT and *ir*-BTPT polymer NPs exhibited a 22- to 40-fold greater phototoxicity against A-549, Sk-Br-3, and MCF-7 human carcinoma cells than the non-polymeric analogue BTPT-OD. The measured photothermal conversion efficiencies ranged from 24 to 27 ± 5%. At the same time, the intracellular ROS generation quantified by the 2′,7′-dichlorodihydrofluorescein diacetate (DCFH-DA) assay was low, allowing us to propose heat-mediated photothermal therapy as a more significant cell death predictor than ROS-mediated photodynamic therapy. This work is one of the first to compare small and polymeric non-fullerene acceptor materials for phototherapy purposes, demonstrating the advantages of using polymers.

## 1. Introduction

Phototherapy is one of the most promising areas of modern, low-invasive cancer treatment. It is based on the use of light-sensitive agents that can generate reactive oxygen species (ROS) or convert light energy into heat when irradiated [1,2]. Due to its high spatial and temporal selectivity, minimal invasiveness, and compatibility with diagnostic methods, phototherapy—including photodynamic therapy (PDT) and photothermal therapy (PTT)—is regarded as an effective alternative to conventional approaches for treating malignant tumors [3,4,5].

One of the key areas of phototherapy development is the creation of new photosensitizers (PSs) that are biocompatible, selectively accumulate in tumor cells, highly photo- and chemically stable [6,7], and absorb effectively in the red and near-infrared (NIR) regions of the spectrum [8,9]. Using NIR light allows for deeper penetration into biological tissues, reducing the destruction of the surrounding tissues and increasing the effectiveness of therapy. However, the therapeutic potential of most traditional PSs (based on porphyrins, phthalocyanines, cyanines, etc.) is significantly reduced due to their absorption in the visible range of the spectrum [10], limited photostability, low solubility in aqueous media, and undesirable aggregation [11,12].

In recent years, research into organic semiconductor materials (OSMs) based on conjugated small molecules and polymers has increased. These materials were originally developed for use in optoelectronic devices, such as organic solar cells [13,14], organic light-emitting devices, and organic field effect transistors [15]. However, OSMs have recently been investigated for use in biomedical applications. These materials offer several advantages, including high photostability, tunable optical and electronic properties, photoluminescence, biocompatibility, and low toxicity [16]. Many OSMs are donor–acceptor (D–A) structures, promoting intramolecular charge transfer (ICT) and reducing the optical bandgap for light absorption in the NIR range of the spectrum. Due to their unique photophysical properties, OSMs are being actively researched for use in PDT and PTT applications [17,18], including in NIR PDT/PTT [19], antibacterial therapy [20,21,22], bioelectronics [23], bioimaging [24,25], and theranostics [26,27]. OSM-based nanoparticles (NPs) are of particular interest due to their nanoscale size, absorption and fluorescence in the visible or NIR regions of the electromagnetic spectrum, biocompatibility, and selectivity [28]. Furthermore, OSMs NPs can accumulate in tumor tissues due to the enhanced permeability and retention (EPR) effect [29], ensuring targeted delivery and enhancing the therapeutic effect.

Recently, low-molecular-weight OSM-based PSs have been demonstrated [30]. For instance, in 2021, Cai et al. demonstrated that TPBT-based NPs, belonging to the Y-series of non-fullerene acceptor (NFA) molecules, exhibited high photothermal conversion efficiency (∼36.5%) and effective ROS generation capacity for combined PTT/PDT in cancer [31]. Small molecules offer a distinct advantage over polymers: they are monodispersed and can be purified using standard techniques. In contrast, polymers exhibit batch-to-batch and scale-dependent variations in molecular weight, which can lead to slight inconsistencies in properties such as NPs size. However, despite the advantages of low-molecular-weight OSMs, they tend to aggregate and generally exhibit limited absorption in the NIR region, rarely exceeding 700 nm. Conjugated polymers, on the other hand, may exhibit improved photostability and colloidal stability without any additional stabilizers, and can incorporate multiple donors and acceptors in a linear arrangement. The latter allows them to demonstrate strong light absorption in the longer wavelength region of the spectrum, enabling deep penetration into biological tissues. Nevertheless, despite the rapidly growing interest in high-performance NIR PSs, research efforts in this field have primarily focused on small molecules, while examples investigating D–A-conjugated polymers are scarce. Moreover, there is a complete absence of studies that directly compare the efficacy of D–A small molecules and their polymeric counterparts. Research in this direction could elucidate fundamental differences between low-molecular-weight chromophores and polymeric analogs in the context of phototherapy, thereby accelerating the development of rational molecular design principles for the next generation of efficient PSs.

## 2. Materials and Methods

### 2.1. Materials

*Tetrakis*(triphenylphosphine) palladium (0) [Pd(PPh_3_)_4_] (LEAPChem), 2,5-*bis*(tributylstannyl)thiophene (SunaTech Inc., Suzhou, China), 2-(5-bromo-3-oxo-2,3-dihydro-1*H*-inden-1-ylidene)malononitrile (SunaTech Inc., Suzhou, China), 2-(5(6)-bromo-3-oxo-2,3-dihydro-1*H*-inden-1-yl)malononitrile (AmBeed, Buffalo Grove, IL, USA), and 1,1-dicyanomethylene-3-indanone were used without further purification. BTPT-OD and 10,11-*bis*(2-octyldodecyl)-10,11-dihydro-[1,2,5]thiadiazolo[3,4-*e*]thieno[2′,3′:4,5]pyrrolo[3,2-*g*]thieno- [3,2-*b*]indole-2,8-dicarbaldehyde (compound 1) were obtained as reported elsewhere [32]. Toluene, chloroform (CHCl_3_), methanol, acetone, and hexane were purified and dried according to the known techniques and then used as solvents. All reactions, unless stated otherwise, were carried out under an inert atmosphere using anhydrous solvents. Toluene was additionally distilled under an argon atmosphere before the reaction. Other reagents used were purchased from commercial sources and used as received.

### 2.2. Synthetic Procedures

2,2′-((2Z,2′Z)-((10,11-*Bis*(2-octyldodecyl)-10,11-dihydro-[1,2,5]thiadiazolo[3,4-*e*] thieno[2′,3′:4,5]pyrrolo[3,2-*g*]thieno[3,2-*b*]indole-2,8-diyl)*bis*(methanylylidene))*bis*(3-oxo-2,3-dihydro-1*H*-indene-2,1-diylidene))dimalononitrile (BTPT-OD). Compound 1 (0.22 g, 0.23 mmol) and 1,1-dicyanomethylene-3-indanone (0.23 g, 1.20 mmol) were dissolved in chloroform (9 mL) in a round-bottom flask under argon. Then, pyridine (0.7 mL) was added, and the mixture was stirred at 60 °C for 10 h. After cooling to room temperature, the reaction mixture was concentrated under reduced pressure. The concentrated mixture was purified using column chromatography on silica gel using dichloromethane/petroleum ether (1/1, *v*/*v*) as the eluent to yield a dark blue solid. The resulting solid was dissolved in a minimal volume of THF, then precipitated with methanol and filtered to yield pure BTPT-OD (0.30 g, 91% yield). ^1^H NMR (250 MHz, CDCl_3_) δ (ppm) = 8.93 (s, 2H), 8.64–8.73 (m, 2H), 7.93–8.02 (m, 4H), 7.73–7.81 (m, 4H), 4.54 (d, *J* = 7.23 Hz, 4H), 2.14–2.02 (overlapping peaks, 2H), 1.25–0.89 (overlapping peaks, 64H), 0.83–0. 75 (overlapping peaks, 12H). ^13^C NMR (75 MHz, CDCl3) δ (ppm) = 187.04, 159.80, 146.89, 145.91, 139.54, 139.48, 137.61, 136.44, 135.34, 135.25, 134.50, 134.04, 125.35, 124.72, 123.35, 122.52, 114.33, 114.26, 114.25, 113.00, 68.66, 54.51, 37.97, 31.45, 31.35, 30.10, 29.37, 29.15, 29.06, 28.98, 28.88, 28.78, 25.40, 22.20, 22.17, 13.68. MALDI-TOF MS for C_80_H_94_N_8_O_2_S_3_: found *m*/*z* 1295.11; calcd. *m*/*z* for [M]^+^ 1294.67. 

2,2′-((2Z,2′Z)-((10,11-*Bis*(2-octyldodecyl)-10,11-dihydro-[1,2,5]thiadiazolo[3,4-*e*] thieno[2′,3′:4,5]pyrrolo[3,2-*g*]thieno[3,2-*b*]indole-2,8-diyl)*bis*(methaneylylidene))*bis*(5-bromo-3-oxo-2,3-dihydro-1*H*-indene-2,1-diylidene))dimalononitrile (monomer 2). Compound 1 (0.1 g, 0.11 mmol) and 2-(5-bromo-3-oxo-2,3-dihydro-1*H*-inden-1-ylidene)malononitrile (0.07 g, 0.24 mmol) were dissolved in 3 mL of chloroform in a round-bottom flask under argon. Then, pyridine (0.2 mL) was added, and the mixture was stirred at room temperature overnight in the dark. After 50 mL of methanol was added, the precipitate was filtered off. The precipitate was chromatographically purified on a silica gel column using petroleum ester/chloroform (2/1, *v*/*v*) as the eluent. After removal of chloroform, the product was reprecipitated in methanol and collected by filtration to yield a dark solid (0.13 g, 84% yield). ^1^H NMR (250 MHz, CDCl_3_): δ 8.89 (s, 2H), 8.51 (d, *J* = 8.3 Hz, 2H), 8.00 (d, *J* = 2 Hz, 2H), 7.81 (dd, *J* = 8.3, 2.0 Hz, 2H), 4.57 (d, *J* = 7.9 Hz, 4H), 2.15 (dq, *J* = 13.2, 6.1 Hz, 2H), 1.28–0.89 (overlapping peaks, 64H), 0.80 (dt, *J* = 14.0, 7.2 Hz, 12H). ^13^C NMR (75 MHz, CDCl_3_): δ (ppm) 185.83, 158.90, 147.23, 146.46, 139.89, 138.36, 138.16, 137.54, 135.89, 135.73, 129.72, 126.76, 121.32, 126.00, 122.20, 114.58, 113.52, 99.91, 72.41, 69.45, 62.48, 55.04, 38.52, 31.86, 31.78, 30.62, 30.59, 29.86, 29.65, 29.58, 29.57, 29.52, 29.45, 29.30, 29.22, 25.93, 25.89, 22.64, 22.62, 22.58, 14.05. MALDI-TOF MS for C_80_H_92_Br_2_N_8_O_2_S_3_: found *m*/*z* 1453.72; calcd. *m*/*z* for [M]^+^ 1453.66.

*r*-BTBT: monomer 2 (0.12 mg, 0.08 mmol), 2,5-*bis*(tributylstannyl)thiophene (0.054 g, 0.08 mmol), and dry toluene (4 mL) were added to a 10 mL double-neck round-bottom flask. The reaction container was purged with argon for 20 min, and then Pd(PPh_3_)_4_ (1.4 mg) was added. The mixture was heated to reflux for 4 h. The reactant was cooled down to room temperature and poured into methanol (100 mL), and filtered. A Soxhlet extraction successively using methanol, acetone, hexane, and chloroform was performed. The polymer r-BTBT with a weight of 91 mg (yield 64%) was recovered as a dark solid from the chloroform fraction by precipitation from methanol, and was filtered and dried under vacuum. GPC: Mn = 10.8 kDa, Mw = 20.6 kDa, Đ = 1.91. ^1^H NMR (CDCl_3_, 250 MHz): δ (ppm) 8.92–8.88 (br, 2H), 8.69–8.51 (br, 2H), 8.13–8.03 (br, 2H), 8.01–7.95 (br, 2H), 7.84–7.79 (br, 2H), 7.60–7.71 (br, 3H), 7.24–7.07 (br, 2H), 4.58–4.50 (br, 4H), 2.23–2.15 (br, 2H), 1.32–0.89 (overlapping peaks, 64H, CH2), 0.86–0.78 (m, 26H).

2,2′-((2Z,2′Z)-((10,11-*Bis*(2-octyldodecyl)-10,11-dihydro-[1,2,5]thiadiazolo[3,4-*e*]thieno[2′,3′:4,5]pyrrolo[3,2-*g*]thieno[3,2-*b*]indole-2,8-diyl)*bis*(methaneylylidene))*bis*(5,6 -bromo-3-oxo-2,3-dihydro-1*H*-indene-2,1-diylidene))dimalononitrile (monomer 3). Compound 1 (0.68 g, 0.72 mmol) and 2-(5(6)-bromo-3-oxo-2,3-dihydro-1*H*-inden-1-yl)malononitrile (0.41 g, 1.51 mmol) were dissolved in 20 mL of chloroform in a round-bottom flask under argon. Then, pyridine (6.5 mL) was added, and the mixture was stirred at room temperature overnight in the dark. After, 200 mL of methanol was added, and the precipitate was filtered off. The precipitate was chromatographically purified on a silica gel column using petroleum ester/chloroform (2/1, *v*/*v*). After removal of chloroform, the product was reprecipitated in methanol and collected by filtration to yield a dark solid (0.84 g, 80% yield). ^1^H NMR (250 MHz, CDCl3): δ (ppm) 8.76 (s, 2H), 7.78 (d, *J* = 8.1 Hz, 2H), 8.88 (s, 2H), 7.88 (m, 4H), 4.56 (d, *J* = 7.9 Hz, 4H), 2.15 (dq, *J* = 13.2, 6.1 Hz, 2H), 1.28–0.89 (overlapping peaks, 64H), 0.80 (dt, *J* = 14.0, 7.2 Hz, 12H). ^13^C NMR (75 MHz, CDCl3): δ (ppm) 185.84, 158.93, 158.73, 147.26, 146.48, 139.89, 138.38, 138.16, 137.55, 135.87, 135.73, 129.72, 126.76, 121.32, 125.96, 126.00, 122.21, 114.58, 113.52, 99.93, 72.42 69.45, 62.48, 55.04, 38.52, 31,86, 31.78, 30.62, 30.59, 29.86, 29.65, 29.58, 29.57, 29.52, 29.45, 29.30, 29.22, 25.93, 25.89, 22.64, 22.62, 22.58, 14.06. MALDI-TOF MS for C_80_H_92_Br_2_N_8_O_2_S_3_: found *m*/*z* 1453.65; calcd. *m*/*z* for [M]^+^ 1453.66. 

*ir*-BTBT: monomer 3 (0.14 g, 0.096 mmol), 2,5-*bis*(tributylstannyl)thiophene (0.064 g, 0.096 mmol) and dry toluene (4.5 mL) were added to a 10 mL double-neck round-bottom flask. The reaction container was purged with argon for 20 min, and then Pd(PPh_3_)_4_ (1.6 mg) was added. The mixture was heated to reflux for 4 h. The reactant was cooled down to room temperature and poured into methanol (100 mL), then filtered. A Soxhlet extraction successively using methanol, acetone, hexane, and chloroform was performed. The polymer *ir*-BTBT with a weight of 83 mg (yield 60%) was recovered as a dark solid from the chloroform fraction by precipitation from methanol, and was filtered and dried under vacuum. GPC: Mn = 8.5 kDa, Mw = 18.2 kDa, Đ = 2.14. ^1^H NMR (CDCl3, 250 MHz): δ (ppm) 8.90–8.89 (br, 2H), 8.68–8.51 (br, 2H), 8.14–8.03 (br, 2H), 8.00–7.96 (br, 2H), 7.84–7.78 (br, 2H), 7.63–7.72 (br, 4H), 7.24–7.06 (br, 2H), 4.57–4.50 (br, 4H), 2.23–2.14 (br, 2H), 1.32–0.89 (overlapping peaks, 68H, CH2), 0.86–0.79 (m, 24H).

Preparation of NPs. 0.5 mL of tetrahydrofuran (THF) solution containing 0.6 mg of either BTPT-OD, *r*-BTPT, or *ir*-BTPT was poured into 4 mL of deionized water with intense stirring, and a colloidal solution was obtained using the nanoprecipitation method [33,34]. THF was removed using dialysis. Hydrodynamic diameters of all obtained NPs were determined using the DLS method.

### 2.3. General Methods

^1^H NMR spectra were recorded at a “Bruker WP-250 SY ”spectrometer (Bruker Corporation, Billerica, MA, USA), working at a frequency of 250.13 MHz and utilizing the CDCl_3_ signal (7.25 ppm) as the internal standard.

Molecular weight characteristics of the polymer were studied by gel permeation chromatography (GPC) using polystyrene standards. The experimental studies were conducted on a Shumadzu apparatus that was equipped with a Smartline RI 2300 refractometer, (KNAUER, Berlin, Germany) utilising THF as an eluent. The chromatographic column, Phenogel 7.8 × 300 mm, was filled with a sorbent that possessed a pore size of 500 Å and a molecular weight of 20 kDa.

UV-Vis absorption spectra were recorded on a SILab u-Violet R (China) spectrophotometer (Beijing Beifen-Ruili Analytical Instrument (Group) Co., Ltd., Beijing, China). Films were cast from CHCl_3_ solutions on glass substrates. All measurements were carried out at room temperature.

Cyclic voltammetry measurements were carried out using solid compact layers of the oligomers, which in turn were made by electrostatically rubbing the materials onto a glassy carbon electrode using IPC-Pro M potentiostat (Volta LiC, Moscow, Russia). Measurements were made in acetonitrile solution using 0.1 M Bu_4_NPF_6_ as supporting electrolyte. The scan rate was 200 mV s^−1^. CV measurements were performed in a standard three-electrode cell equipped with a glassy carbon working electrode (s = 2 mm^2^), a platinum plate as the counter electrode, and SCE (saturated calomel electrode) as the reference electrode. The highest occupied molecular orbital (HOMO) and the lowest unoccupied molecular orbital (LUMO) energies were evaluated using the first standard oxidation (φ_ox_) and reduction (φ_red_) potentials obtained from CV experiments as E(HOMO) = –e(φ_ox_ + 4.40) (eV) and E(LUMO) = –e(φ_red_ + 4.40) (eV), where is the elementary charge [35].

Dynamic Light Scattering (DLS) and ζ-Potential Measurements. Hydrodynamic diameters of the NPs in water were determined by DLS at 25 °C using a Microtrac Zetatrac instrument (Microtrac Inc., Montgomeryville, PA, USA) equipped with a 4 mW He−Ne solid-state laser with a 780 nm wavelength. Scattered light was detected at 180° in a controlled reference self-beating mode, and the particle size was calculated from the power spectrum analysis of the Doppler-shifted signal over several runs. For every tested sample, 3 runs, each of 60 s duration, were performed. The sphere-equivalent hydrodynamic diameter of the NPs was calculated from the particle diffusion coefficient via the Stokes−Einstein equation, using a solution viscosity for liquid media at 25 °C. Electrophoretic mobility of the NPs was measured in liquid media using a Microtrac Zetatrac instrument by applying AC/DC voltage to the built-in sample cell electrodes to determine the magnitude and sign of particle ζ-potential. ζ-Potential was calculated from the electrophoretic mobility via the Smoluchowski equation. All measurements and data analysis were performed using NanoFLEX Software version 11.1.07.

Nanoparticle stability Assay. BTPT-OD, *r*-BTPT, and *ir*-BTPT NPs were placed in sealed disposable cuvettes for DLS measurements and incubated at room temperature. At predetermined time intervals, the hydrodynamic size of NPs was measured by DLS. Experiments were performed in triplicate. The stability of the NPs was determined by the decrease in the absorption spectrum maximum over time. Absorption spectra were measured using a SILab u-Violet R spectrophotometer (China) in standard 10 mm photometric quartz cuvettes. Diluted aqueous solutions of NPs were placed in cuvettes, which were then closed with polytetrafluoroethylene (PTFE) caps and sealed with paraffin tape. All measurements were carried out at room temperature.

Absorption spectroscopy. The absorption spectra were recorded with a SILab u-Violet R spectrophotometer (China) in the standard 10 mm photometric quartz cuvette. The absorption spectra were recorded using THF solutions with concentrations of 10^−5^ M. The absorption spectra of the NPs dispersions of the corresponding compounds were recorded using distilled water as a reference sample. All measurements were carried out at room temperature.

Atomic Force Microscopy (AFM) data. The samples for AFM investigation were obtained on mica substrates (Perspective technologies center, Moscow, Russia) in two ways. Prior to deposition, the upper layer of mica substrates was removed using tape. For the first way, an NPs solution drop of 20 μL was placed on the mica substrate, and the substrate was stored in a humid atmosphere for 30 min to prevent evaporation. Then, the substrate was rinsed with deionized water and dried in a nitrogen flow. For the second way, an NPs solution drop of 10 μL was evaporated from the substrate at room temperature.

Cytotoxicity study. Human breast adenocarcinoma Sk-Br-3 and MCF-7 and human lung carcinoma A-549 cells were seeded in 96-well plates (10^5^ cells per well) in complete DMEM medium. The next day, the medium in some wells was replaced with 100 μL of fresh medium containing BTPT-OD, *r*-BTPT, and *ir*-BTPT NPs in the concentration range of 2–26 μM (total TRI points + control). Cells were incubated with NPs for 1.5 h, after which they were irradiated at a 730 nm wavelength for 12 min (light dose ~30 J/cm^2^); non-irradiated cells were used as a control. Cell viability was assessed after 72 h using the MTT method. For this purpose, MTT solution (0.5 mg/mL) was added to each well. After 2 h, the medium was removed, and 100 μL of DMSO (99%) was added. Absorbance was recorded at 565 nm using an Infinite M Nano reader (Tecan Group Ltd., Männedorf, Switzerland). The IC_50_ values were determined using GraphPad Prism 10.3.0 software.

Intracellular ROS generation study by flow cytometry (DCFH-DA staining). Human breast carcinoma cancer Sk-Br-3 cells were seeded in 6-well slides (~250,000 cells per well) in complete DMEM medium. The next day, the medium in each well was replaced with fresh medium containing BTPT-OD, *r*-BTPT, and *ir*-BTPT NPs at a concentration of 8 μM for BTPT-OD and 0.4 μM for *r*-BTPT and *ir*-BTPT NPs. The cells were incubated for 5 min, and 1 and 2 h, after which the 2′,7′-dichlorofluorescin diacetate (DCFH-DA) (1 μM) was added to each well for 30 min in serum-free PBS (pH 7.4). The cells were irradiated at a wavelength of 730 nm with a 30 J/cm^2^ light dose. The cells were then harvested with a trypsin solution (0.25%), washed with saline, and evaluated by flow cytometry using the FITC channel; at least 20,000 events were analyzed for each sample.

Intracellular ROS generation study by flow cytometry (CellROX™ Deep Red staining). Human breast carcinoma cancer Sk-Br-3 cells were seeded in 24-well slides (~100,000 cells per well) in complete DMEM medium. The next day, the medium in each well was replaced with fresh medium containing BTPT-OD, *r*-BTPT, and *ir*-BTPT NPs at a concentration of 20, 10, and 1 μM for BTPT-OD and 0.25, 0.5, and 1 μM for *r*-BTPT and *ir*-BTPT NPs. The cells were incubated for 2 h, after which the CellROX™ Deep Red Reagent (2.5 μM) was added to each well for 30 min in complete DMEM. The cells were irradiated at a wavelength of 730 nm with a 30 J/cm^2^ light dose. The cells were then harvested with a trypsin solution (0.25%), washed with saline, and evaluated by flow cytometry using the APC channel; at least 10,000 events were analyzed for each sample.

Intracellular accumulation study by fluorescence spectroscopy. Human breast carcinoma cancer Sk-Br-3 cells were seeded in 24-well slides (~100,000 cells per well) in complete DMEM medium. The next day, the medium in each well was replaced with fresh medium containing BTPT-OD, *r*-BTPT, and *ir*-BTPT NPs at a concentration of 8 μM for BTPT-OD and 0.5 μM for *r*-BTPT and *ir*-BTPT NPs. The cells were incubated for 30 min and 2 h and then harvested with a trypsin solution (0.25%), washed with saline, and evaluated by fluorescence spectroscopy method at 670 nm excitation wavelength.

Photothermal data for NPs and PCE calculation. The aqueous solutions of BTPT-OD, *r*-BTPT, and *ir*-BTPT NPs with different concentrations were continuously exposed to a 730 nm laser with the power density of 0.06 W/cm^2^ for 6 min, and then the NPs dispersions were allowed to cool to room temperature. The temperature of the dispersions was monitored during the whole process.

Thermal measurements were conducted on the 0.1 mL aliquots of water solutions of the under-study NPs, and water was used as a reference. Substances in 1.5 mL Eppendorf were subsequently placed in a holder above optical table level and illuminated at middle-column height with collimated LED light (730 nm, 0.06 W/cm^2^) until temperature stabilization with subsequent cooling to room temperature (22 °C). Temperatures were measured with an IR camera Xenics Gobi-384-GigE-7098 (Xenics, Leuven, Belgium) placed perpendicular to both the laser beam and Eppendorf at a distance of ~25 cm, so that Eppendorf was imaged clearly at a rate of 10 fps. Numerical results were then gathered with Xeneth v2.6.0.309 software. Thermal coefficients were evaluated in the following way, adapted from [36] for the specific case of Eppendorf-based measurements.

## 3. Results

### 3.1. Synthesis

This work provides a thorough comparison of three OSMs with a D–A molecular motif: a small molecule BTPT-OD and its two polymer derivatives, the regular *r*-BTPT and the irregular *ir*-BTPT. The polymers *r*-BTPT and *ir*-BTPT differ primarily in their molecular architecture rather than their chemical composition. This molecular regularity can significantly influence the physicochemical properties of both the bulk material and derived NPs. For instance, regular NFA polymers with a 5-substituted (3-oxo-2,3-dihydro-1*H*-inden-1-ylidene)malononitrile acceptor moiety are known to exhibit a red-shifted absorption spectrum compared to their irregular counterparts [37]. Furthermore, such structurally regular NFA polymers tend to form more favorable molecular packing, which enhances charge generation following photoexcitation [38]. Conversely, the increased structural order in regular polymers also predisposes them to stronger aggregation tendencies compared to irregular polymers, a phenomenon that can be exacerbated by higher molecular weight [39]. We therefore hypothesized that the structural regularity of the polymer backbone would be a determinant of NPs phototherapeutic performance and designed an experiment to test this. The comparison may help reveal how the transition from an A–D–A small-molecule structure to regular and irregular polymer chains affects parameters such as absorption, photostability, thermal conversion, ROS generation capacity, and NPs formation and properties, which are key to phototherapeutic efficacy. The resulting data will provide a valuable foundation for developing optimized, next-generation OSM-based agents for cancer phototherapy.

**Figure 1:**
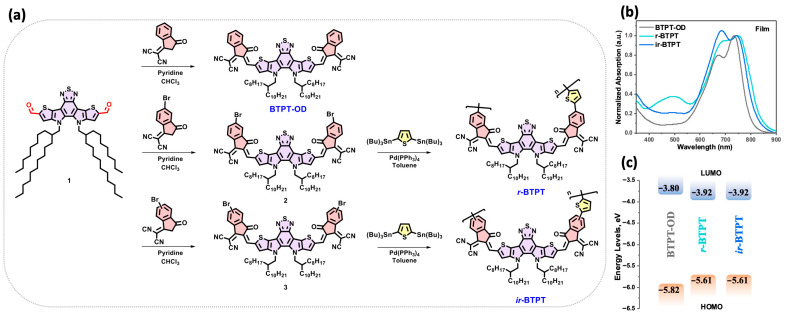
(**a**) Synthesis of the investigated compounds: small molecule BTPT-OD and polymeric derivatives *r*-BTPT and *ir*-BTPT; (**b**) absorption spectra in the thin films; (**c**) energy level diagram of the frontier molecular orbitals.

Figure 1 illustrates the synthesis of the target small molecule and polymeric materials. The small-molecule acceptor BTPT-OD [32] and monomers 2 and 3 were obtained *via* Knoevenagel condensation of dicarbaldehyde 1 with the corresponding indan-1-one-3-dicyanovinyl derivatives. The products were obtained in high yields (90%, 84%, and 80%, respectively). Further, monomers 2 and 3 were subjected to Stille cross-coupling with 2,5-*bis*(tributylstannyl)thiophene under standard conditions. The cross-coupling reactions were performed under reflux for four hours, yielding the target polymers *r*-BTPT and *ir*-BTPT with good yields of 64% and 60%, respectively.

All synthesized compounds exhibited good solubility in common organic solvents, including THF, dichloromethane, chloroform, and toluene, at room temperature. Molecular weight parameters were determined by gel permeation chromatography (GPC) calibrated against polystyrene standards. The number-average molecular weights (Mn), weight-average molecular weights (M_w_), and polydispersity indices (Đ) are summarized in Table 1.

### 3.2. Optical and Electrochemical Properties

The optical properties of the three synthesized compounds were investigated using absorption spectroscopy in thin films (Figure 1b and Table 1). The compounds exhibited intense absorption bands in the 500–840 nm region, typically associated with ICT transitions or mixed ICT/π–π * character arising from their D-A molecular architecture [40]. Although the three materials display comparable absorption maxima, the polymers show markedly broadened and bathochromically shifted spectral profiles compared to BTPT-OD. This behavior reflects the extended π-conjugation and enhanced interchain interactions inherent to the polymeric backbones. As a result, the optical band gaps (*E*_g_^Opt^), derived from the onset of the film absorption spectra in solution and in film, follow the expected trend: the polymers possess narrower *E*_g_^Opt^ than the small molecule.

The electrochemical properties were further evaluated using cyclic voltammetry (CV) to determine the oxidation (*φ*_ox_) and reduction (φ_red_) potentials and to estimate the highest occupied molecular orbital (HOMO) and lowest unoccupied molecular orbital (LUMO), as well as the resulting electrochemical bandgap (*E*_g_). All compounds displayed irreversible redox processes, with electron uptake primarily localized on the electron-deficient structural motifs and oxidation occurring predominantly on the electron-rich segments. In agreement with the optical measurements, the polymers exhibit a narrower *E*_g_ relative to the small molecule, accompanied by lower-lying LUMO energies and elevated HOMO energies (Figure 1c). The close correspondence between the optical and electrochemical band gaps confirms the internal consistency of the measurements and underscores the impact of conjugation extension on tuning the electronic structure of the polymers.

### 3.3. Preparation and Characterization of Nanoparticles

**Figure 2:**
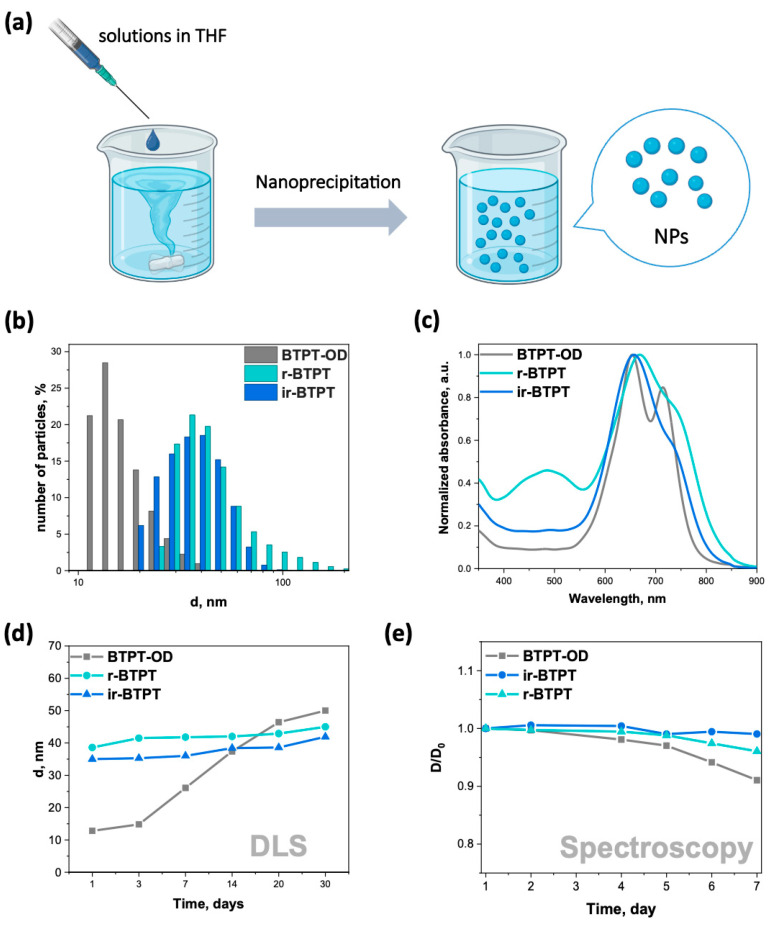
(**a**) Illustration of NFA NPs preparation through nanoprecipitation; (**b**) size distribution of BTPT-OD, *r*-BTPT, and *ir*-BTPT NPs obtained by DLS; (**c**) normalized absorption spectrum of BTPT-OD, *r*-BTPT, and *ir*-BTPT NPs dispersed in water; (**d**) kinetics of BTPT-OD, *r*-BTPT, and *ir*-BTPT NPs size growth obtained by DLS; (**e**) dependence of the relative density at the wavelength of maximum absorption of BTPT-OD, *r*-BTPT, and *ir*-BTPT NPs on time in water; D/D_0_—dependence of the relative density at the wavelength of maximum absorption on time.

Since most OSMs are hydrophobic, they must be converted into a water-compatible form for biomedical applications. This can be achieved through the self-assembly of OSMs into NPs. The resulting BTPT-OD, *r*-BTPT, and *ir*-BTPT NPs in water were prepared using the nanoprecipitation method from the THF solution, followed by dialysis (Figure 2a). This method relies on the formation of NPs through a rapid change in solvent polarity, which encourages π-π packing and hydrophobic interactions [41,42]. The average hydrodynamic diameters (*d*_av_) of the resulting NPs were determined using dynamic light scattering (DLS) (Figure 2b). The average diameter of the BTPT-OD NPs was 13 nm, while the average diameters of the polymeric *r*-BTPT and *ir*-BTPT NPs were 35 and 39 nm, respectively (Figure 2b). These sizes are particularly suitable for biomedical applications, especially phototherapy, since particles with a diameter of less than 10 nm are often rapidly excreted by the kidneys, while larger particles (>200 nm) may have problems with extravasation and may be captured by the macrophage system [43]. The size range of 20–150 nm is considered to be optimal for passive accumulation via the EPR effect [44] and biomedical applications, as such NPs exhibit reduced clearance through the liver and kidneys and have a longer circulation time *in vivo* [45,46]. The smaller diameter of non-polymeric BTPT-OD NPs can be explained by the structure of the molecule. A short molecular backbone and a lower molecular weight allow for denser π-π packing and the formation of smaller aggregates during nanoprecipitation. In contrast, polymeric conjugated materials tend to form larger NPs due to their higher molecular weight and polymer chain entanglements. This leads to an increased hydrodynamic diameter upon self-assembly. It is known that the nanoprecipitation process is influenced by molecular weight, polymer chain flexibility, and intermolecular interactions, which govern the nucleation and growth phases impacting final nanoparticle size [47,48].

Zeta potential measurements confirmed that NPs carried a net negative surface charge, with values of −12,9, −15,5, and −17,9 mV for BTPT-OD, *r*-BTPT, and *ir*-BTPT NPs, respectively, indicating the dispersions had moderate electrostatic stability. NPs with high positive charges are rapidly removed by macrophages [49], while NPs with high negative charges tend to be absorbed and cleared by the liver, spleen, or other parts of the reticuloendothelial system (RES). Neutral and moderately negative NPs typically exhibit prolonged circulation times, although their transmembrane transport may be hindered by electrostatic repulsion. Therefore, the slightly negative surface charge of the obtained NPs is advantageous, as it supports colloidal stability while avoiding rapid RES uptake, thereby promoting more favorable *in vivo* circulation [50].

The normalized absorption spectrum of the aqueous dispersion of NPs shows absorption of light with a pronounced band in the red and NIR region (Figure 2c). The spectra of NPs in terms of shape and position of bands practically coincide with the spectra of thin films, reflecting the similar nature of intermolecular interactions in ordered aggregated structures.

The stability of NPs intended for use in biomedical applications, particularly phototherapy, is critically important. The dimensional stability of BTPT-OD, *r*-BTPT, and *ir*-BTPT NPs was verified by measuring their hydrodynamic diameter in water over time using DLS (Figure 2d) and absorption spectroscopy (Figure 2e and Figure S1). Based on the DLS data, the NPs of BTPT-OD are significantly less stable than the NPs of both polymers. Over 30 days, the average hydrodynamic diameter of BTPT-OD NPs increased from 13 to 39 nm. In contrast, the sizes of *r*-BTPT and *ir*-BTPT NPs increased by only 9 and 10 nm, respectively (Figure 2d). The flexible, long-chain structure of the polymers ensures stable colloidal dispersion with minimal aggregation due to steric hindrance and electrostatic repulsion [51]. In contrast, small-molecule-based NPs self-assemble via weaker hydrophobic and π-π interactions, resulting in increased aggregation and growth in size over time.

It is worth noting that a similar trend of increased colloidal stability over time was confirmed for *r*-BTPT and *ir*-BTPT in saline/fetal bovine serum (FBS) mixtures containing 1% and 10% FBS compared to BTPT-OD (Figure S2). Furthermore, the hydrodynamic diameters of fresh BTPT-OD and *r*-BTPT NPs increased significantly in serum solution compared to water. BTPT-OD NPs in water had a d_av_ of 13 nm; in 1% and 10% FBS solutions, their sizes were 28 and 35 nm, respectively. The same was observed for *r*-BTPT NPs: their d_av_ was 39 nm in water and 49 and 51 nm in 1% and 10% FBS solutions, respectively. Therefore, the difference in size between the polymer NPs in serum solutions increased; however, both polymer dispersions remained stable in a physiological environment for two days, increasing in size by only 5–9 nm. In contrast, BTPT-OD NPs exhibited more active aggregation, growing by over 30 nm within 2 days (Figure S2).

**Figure 3:**
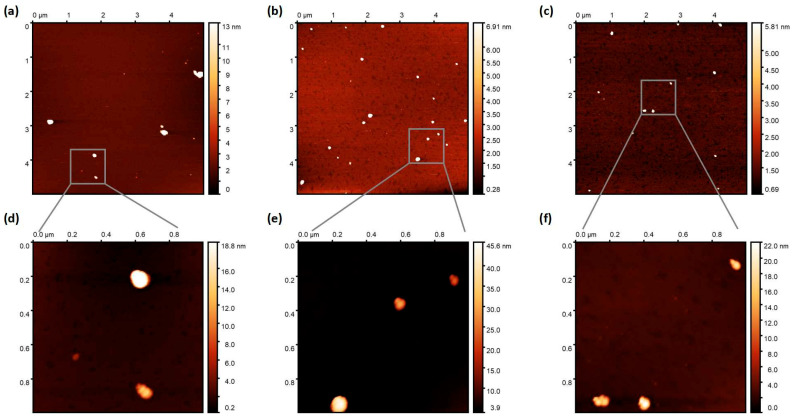
AFM images (topography) of BTPT-OD (**a**), (**d**) *r*-BTPT (**b**,**e**), and *ir*-BTPT (**c**), (**f**) with scan size of 5 × 5 µm (**a**–**c**) and 1 × 1 µm (**c**–**e**).

The trends from DLS data are in agreement with AFM data (Figure 3). To obtain the AFM data, NPs were deposited onto mica substrates (for details, please see the Experimental Section). It should be noted that the obtained NPs dispersions are rather diluted; therefore, there were 5–15 NPs at the 5 × 5 µm scan. Using bigger scans is not advisable, since the particle diameter will be measured with a greater margin of error. As there were not many NPs, the average NPs size was obtained by averaging 2–3 scans (10–20 particles). The average NPs sizes were found to be 60 ± 10 nm, 84 ± 15 nm, and 85 ± 19 nm for BTPT-OD, *r*-BTPT, and *ir*-BTPT NPs, respectively. It should be noted that these sizes are upper estimates, as it is clear from 1 × 1 µm scans that some particles represent aggregations of several particles. In the case of *r*-BTPT and *ir*-BTPT, the size of the smaller particles is around 35 nm, which is consistent with DLS data. BTPT-OD NPs have the greatest tendency to aggregate (the scan shows a particle consisting of five particles). According to AFM, the size of the smaller BTPT-OD particles is also approximately 30 nm, compared to the approximately 13 nm obtained by DLS for the as-prepared NPs dispersion. This also indicates their greater tendency to aggregate. It is worth mentioning that aggregation worsens during solution evaporation (Figure S3).

### 3.4. In Vitro Experiments

**Figure 4:**
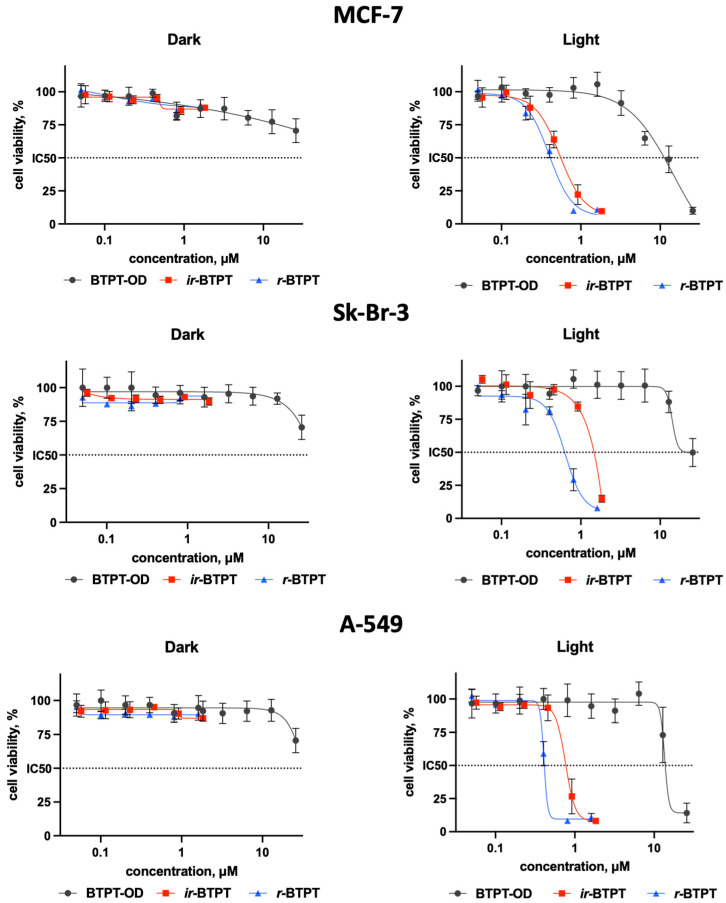
Viability of human breast Sk-Br-3, MCF-7, and lung adenocarcinoma A-549 cells depending on the presence of BTPT-OD, *r*-BTPT, and *ir*-BTPT NPs (μM) in the dark and light (730 nm, 30 J/cm^2^) conditions, MTT assay, 72 h incubation; IC_50_—half max. inhibitory concentration (dashed line).

The antiproliferative activity of BTPT-OD, *r*-BTPT, and *ir*-BTPT NPs was investigated using human breast Sk-Br-3 and MCF-7 and human lung adenocarcinoma A-549 cells under light and dark conditions (Figure 4). All NPs demonstrated negligible cytotoxicity in the absence of irradiation, indicating their good biocompatibility and safety profile in the dark. Upon NIR irradiation (730 nm, 30 J/cm^2^), a pronounced difference in phototoxicity was observed between the low-molecular BTPT-OD and its polymeric analogues *r*-BTPT and *ir*-BTPT. The polymer-based NPs showed higher phototherapeutic efficacy, which is reflected in their lower IC_50_ values. For example, the IC_50_ values on A-549 cells for *ir*-BTPT and *r*-BTPT NPs exceeded those for BTPT-OD by 22 and 40 times, respectively (Table 2). Similar differences in IC_50_ values on Sk-Br-3 and MCF-7 cells were observed for *ir*-BTPT and *r*-BTPT NPs compared to BTPT-OD NPs: 20- and 27-fold for MCF-7 cells, and 20- and 41-fold for Sk-Br-3 cells, respectively. It is worth noting that the IC_50_ values for *r*-BTPT NPs were higher than those for *ir*-BTPT NPs (Table 2). The slightly greater phototoxicity of the *r*-BTPT NPs may be due to the longer wavelength of its absorption that allows it to perform better under 740 nm diode irradiation compared to *ir*-BTPT NPs.

The intracellular accumulation of the NPs was studied using the fluorescence spectroscopy method. It was observed that polymeric NPs accumulated in cells more efficiently than the non-polymeric BTPT-OD NPs. Among the polymeric systems, the regular *r*-BTPT NPs demonstrated noticeably higher intracellular uptake compared to the irregular *ir*-BTPT NPs (Figure S4, ESI). These findings correlated with the cytotoxicity data that demonstrated the superior toxicity of r-BTPT NPs. Polymeric *r*-BTPT and *ir*-BTPT NPs have more controllable and stable sizes (Figure 2), which may facilitate endocytosis [52]. Non-polymeric BTPT-OD NPs, on the other hand, rapidly aggregate, reducing the efficiency of cellular uptake. Moreover, it is known that low-molecular compounds could be effluxed from the cytoplasm by P-gp proteins and other multidrug-resistance related proteins [53].

The intracellular production of ROS following irradiation was quantified using -DCFH-DA and CellROX™ Deep Red as trapping agents. It was found that NPs generated no detectable ROS in Sk-Br-3 cells even under harsh (30 J/cm^2^) 730 nm irradiation (Figures S5 and S6, ESI). Based on the knowledge that phototherapy has two options, namely ROS-mediated PDT and heat-mediated PTT, only a slight involvement of PDT in the total phototoxicity could be proposed. Therefore, PTT could be discussed as the major toxicity pathway for the studied preparations.

### 3.5. Photothermal Ability

**Figure 5:**
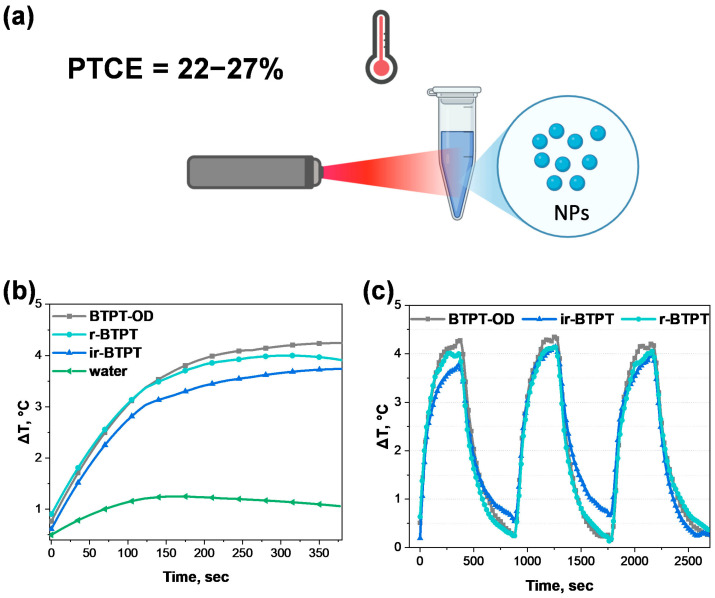
Photothermal conversion efficiency determination. (**a**) General scheme of photothermal properties evaluation of BTPT-OD, *r*-BTPT, and *ir*-BTPT NPs; (**b**) photothermal response of BTPT-OD, *r*-BTPT, and *ir*-BTPT NPs (20 μg/mL) and water under 730 nm laser irradiation (0.06 W/cm^2^) for 6 min. Heating is shown relative to a room temperature of 22 °C; (**c**) temperature elevation curves of 3 heating–cooling cycles of 20 μg/mL BTPT-OD, *r*-BTPT, and *ir*-BTPT NPs under 730 nm LED (0.06 W/cm^2^).

It is assumed that the key role in the photoinduced toxicity mechanism of the studied NPs in relation to cancer cells is played by controlled photoconversion of light into heat. The photothermal conversion capacity of the NPs was evaluated in an aqueous solution under the action of NIR irradiation (Figure 5a). Heating curves of the NPs in an aqueous solution, compared to pure distilled water as a control, were measured under low-intensity excitation by a light-emitting diode (peak wavelength 730 nm, full width at half maximum 30 nm, power density 0.06 W/cm^2^) for 6 min (Figure 5b). As can be seen in Figure 5b, the temperature profiles show that the NPs heat up rapidly during 6 min of irradiation. To evaluate the thermal stability of the NPs, successive heating and cooling cycles were performed by turning the diode on for 6 min of irradiation and then turning it off. Notably, even after three heating–cooling cycles (activating the diode for 6 min, then deactivating it until thermalization to ambient temperature), the heating curves retained the same shape and amplitude. This indicates that there was no photodegradation or structural change in the NPs during cyclic irradiation (Figure 5c), which is an important requirement for photothermal agents intended for repeated or prolonged therapeutic use.

The thermal conversion coefficient was evaluated using a standard method based on solving the heat balance equation for heating and cooling curves under the approximation of a linear cooling rate with respect to temperature. Based on these data, the lower bound for photothermal conversion efficiency (PTCE) of the NPs was estimated to be 24 ± 5% for BTPT-OD NPs, 22 ± 5% for *r*-BTPT NPs, and 27 ± 5% for *ir*-BTPT NPs. These PTCE values are within the typical range for polymeric photothermal nanomaterials and are comparable to those of DOX-PPy@PNAs (20%) under 808 nm irradiation, which has demonstrated successful application in synergistic PTT/chemotherapy [54]. While these values remain lower than those of the most advanced polymeric nanosystems, for which calculated PTCE can reach 40–60% [55], BTPT-OD, *r*-BTPT, and *ir*-BTPT NPs exhibit stable and reproducible photothermal activity under low-intensity irradiation. This confirms their practical applicability as reliable, thermally stable photothermal agents. Further fine molecular optimization could enhance the efficiency of these systems even further.

## 4. Conclusions

This study presents the first direct comparison between a structurally similar D-A small-molecule NFA and its polymer counterparts for cancer phototherapy. The transition from a small molecule BTPT-OD to regular *r*-BTPT and irregular *ir*-BTPT polymers significantly improved key properties that determine the efficacy of phototherapeutic agents, including increased absorption in the red and NIR regions, formation of more stable NPs with controlled size, higher cellular uptake, and greater phototoxicity of the polymeric NPs compared to BTPT-OD. Based on the phototoxicity and intracellular ROS analysis data, the leading role of PTT, but not PDT, could be proposed as a major cell death pathway. This highlights the advantages of polymeric acceptors for NIR cancer therapy. Although this work was not intended to compare the system with other available PTT agents with superior photothermal performance, the obtained data demonstrate that polymerizing NFA is a promising approach for creating effective, next-generation photothermal nanomaterials.

Going forward, it is important to expand this study with *in vivo* experiments, including an assessment of the biodistribution and pharmacokinetics of polymer NPs, as well as visualizing the photothermal effect using thermal imaging. Additionally, there is significant interest in exploring the possibilities of combination therapy with conjugates based on similar conjugated polymer systems. One example is integrating PTT with chemotherapy to enhance the antitumor response and reduce the required therapeutic doses.

## Figures and Tables

**Table 1:** Optical, electrochemical, and molecular mass data of BTPT-OD, *r*-BTPT, and *ir*-BTPT.

Compound	*UV−vis* Absorption			CV Data					Molecular Weight Distribution		
	Film			*φ*_ox_, V	*φ*_red_, V	HOMO, eV	LUMO, eV	*E*_g_, eV	M_N_, kDa	M_W_, kDa	Đ
	*λ*_max_, nm	λ_onset_, nm	*E*_g_^Opt^, eV								
BTPT−OD	734	801	1.55	+1.42	−0.60	−5.82	−3.80	2.02	-	-	-
*r*−BTPT	747	848	1.46	+1.21	−0.48	−5.61	−3.92	1.69	12.1	20.6	1.71
*ir*−BTPT	746	834	1.49	+1.21	−0.48	−5.61	−3.92	1.69	8.5	18.2	2.13

Notes: λ_max_ is the absorption spectrum maximum; λ_onset_ is the absorption spectrum edge; E_g_^Opt^ is the optical bandgap value; φ_ox_ is an electrochemical oxidation potential value; φ_red_ is an electrochemical reduction potential value; *E*_g_ is the electrochemical bandgap value.

**Table 2:** The IC_50_ values for the viability of human breast Sk-Br-3, MCF-7 and lung adenocarcinoma A-549 cells after incubation with BTPT-OD, *r*-BTPT, and *ir*-BTPT NPs.

Compound	A-549 Cells		MCF-7 Cells		Sk-Br-3 Cells	
	IC_50_, µM		IC_50_, µM		IC_50_, µM	
	Dark	Light	Dark	Light	Dark	Light
BTPT-OD	>25.72	16.49 ± 2.85	>25.72	10.60 ± 2.02	>25.72	25.59 ± 7.46
*ir*-BTPT	>1.83	0.76 ± 0.10	>1.83	0.54 ± 0.11	>1.83	1.29 ± 0.12
*r*-BTPT	>1.61	0.41 ± 0.09	>1.61	0.40 ± 0.08	>1.61	0.63 ± 0.17

Notes: IC_50_—half max. inhibitory concentration.

## Data Availability

The original contributions presented in this study are included in the article/Supplementary Materials. Further inquiries can be directed to the corresponding author.

## Supplementary Materials

The following supporting information can be downloaded at https://www.mdpi.com/article/10.3390/polym17243304/s1, Table S1. Photothermal coefficient values calculated for BTPT-OD, r-BTPT, and ir-BTPT NPs (20 μg/mL); Figure S1. Absorption spectra of BTPT-OD, r-BTPT, and ir-BTPT NPs in water for 7 days; Figure S2. Kinetics of BTPT-OD, r-BTPT, and ir-BTPT NPs size growth in a mixture of saline/FBS 1% (a) and 10% (b) obtained by DLS. Figure S3. AFM images (topography) of r-BTPT obtained by evaporation of 10 µL solution in a mica substrate; Figure S4. Intracellular accumulation of BTPT-OD, *r*-BTPT, and *ir*-BTPT NPs in human breast adenocarcinoma Sk-Br-3, 10 μM and 0.5 μM for BTPT-OD and *r*-BTPT and *ir*-BTPT NPs, respectively, 30 min and 2 h incubation. The accumulation was measured in the cell lysate in DMSO, ex. 670 nm, em. 850 nm. The cell lysate data were normalized to the fluorescence of the initial suspensions in DMSO; data are the mean ± SEM; Figure S5. ROS generation of BTPT-OD, *r*-BTPT, and *ir*-BTPT NPs in human breast carcinoma Sk-Br-3 cells evaluated by DCFDH staining. The concentration was 8 μM for BTPT-OD and 0.4 μM for *r*-BTPT and *ir*-BTPT NPs (near IC_50_ value), 5 min, 1 h, and 2 h incubation. Cells were irradiated with 730 nm for a total of 30 J/cm^2^ light dose. Primary flow cytometry curves for 10 µg/mL (a) and mean fluorescent signal for all concentrations (b). Flow cytometry data, 20,000 events in each sample, data are the mean ± SD; Figure S6. ROS generation of BTPT-OD, *r*-BTPT, and *ir*-BTPT NPs in human breast carcinoma Sk-Br-3 cells evaluated by CellROX™ Deep Red staining. The concentration was 1 μM and 0.25 μM (sub-IC_50_ value), 10 μM and 0.5 μM (near IC_50_ value), and 20 μM for BTPT-OD and 1 μM for *r*-BTPT and *ir*-BTPT NPs (over IC_50_ value), respectively, after 2 h incubation. Cells were irradiated with 730 nm for a total 30 J/cm^2^ light dose. Primary flow cytometry curves for 10 µg/mL (a) and mean fluorescent signal for all concentrations (b). Flow cytometry data, 10,000 events in each sample, data are the mean ± SD.

## Abbreviations

The following abbreviations are used in this manuscript:

ROS	Reactive oxygen species
PDT	Photodynamic therapy
PTT	Photothermal therapy
PSs	Photosensitizers
DCFH-DA	2′,7′-dichlorodihydrofluorescein diacetate
NIR	Near-infrared
OSMs	Organic semiconductor materials
ICT	Intramolecular charge transfer
NPs	Nanoparticles
NFA	Non-fullerene acceptor
EPR	Enhanced permeability and retention
D-A	Donor-acceptor
GPS	Gel permeation chromatography
THF	Tetrahydrofuran
CV	Cyclic voltammetry
DLS	Dynamic light scattering
RES	Reticuloendothelial system
PTCE	Photothermal conversion efficiency
