# Supplementary material for: From Small Molecules to Polymers: Developing Non-Fullerene Acceptors for Efficient NIR Photothermal Cancer Therapy
Source: Polymers (Basel). 2025 Dec 13;17(24):3304. doi: 10.3390/polym17243304 (PMC12736452; doi:10.3390/polym17243304)
Supplement: Supplementary file 1 [file polymers-17-03304-s001.zip › polymers-4028809-supplementary.pdf]

ELECTRONIC SUPPORTING INFORMATION

From Small Molecules to Polymers: Developing Non-Fullerene Acceptors for Efficient NIR Photothermal Cancer Therapy

Yulia A. Isaeva[1], Elizaveta D. Blagodarnaia[1], Anastasia A. Vetyugova[1], Maxim E. Stepanov[2], Liya A. Poletavkina[1], Ivan V. Dyadishchev[1], Askold A. Trul[1], Tatyana V. Egorova[1,3], Roman A. Akasov[1,3] and Yuriy N. Luponosov[1]*

[1]Enikolopov Institute of Synthetic Polymeric Materials of the Russian Academy of Sciences, Profsoyuz-naya St. 70, Moscow 117393, Russia

[2]Moscow Pedagogical State University, Malaya Pirogovskaya St. 29/7, building 1, Moscow 119991, Russia

[3] Department of Biochemistry, Petrovsky Medical University, Moscow 119435, Russia

*Correspondence: luponosov@ispm.ru

1. Nanoparticles properties

Table S1. Photothermal coefficient values calculated for BTPT-OD, *r*-BTPT and *ir*-BTPT NPs (20 µg/mL)

Compound	OD at 730 nm	Thermal coefficient, %
BTPT-OD	1,28	24 ± 5
r-BTPT	0,68	27 ± 5
ir-BTPT	0,77	22 ± 5

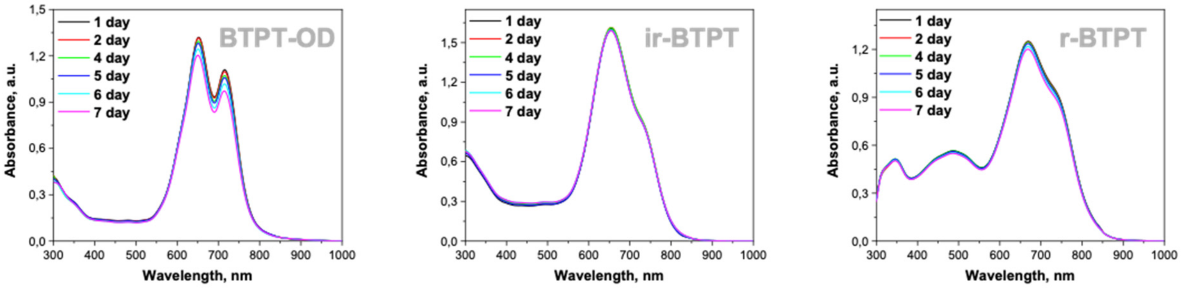

Figure S1. Absorption spectra of BTPT-OD, *r*-BTPT, and *ir*-BTPT NPs in water for 7 days.

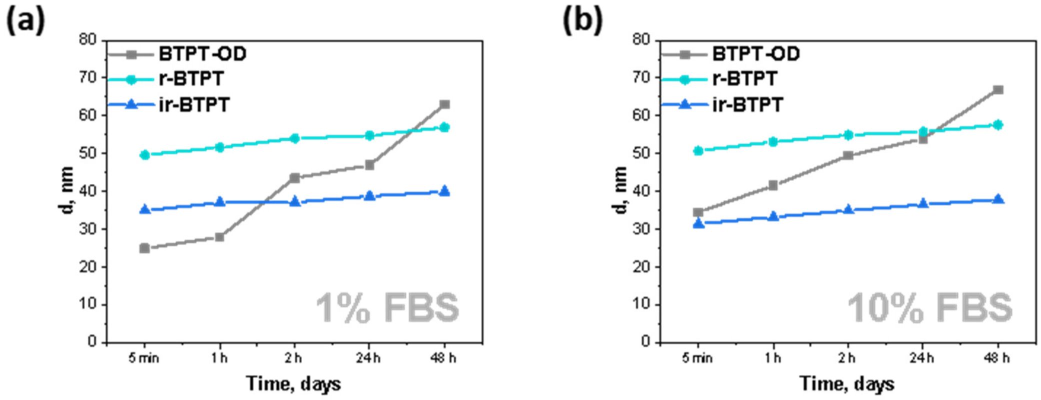

Figure S2. Kinetics of BTPT-OD, r-BTPT and ir-BTPT NPs size growth in a mixture of saline/FBS 1% (a) and 10% (b) obtained by DLS.

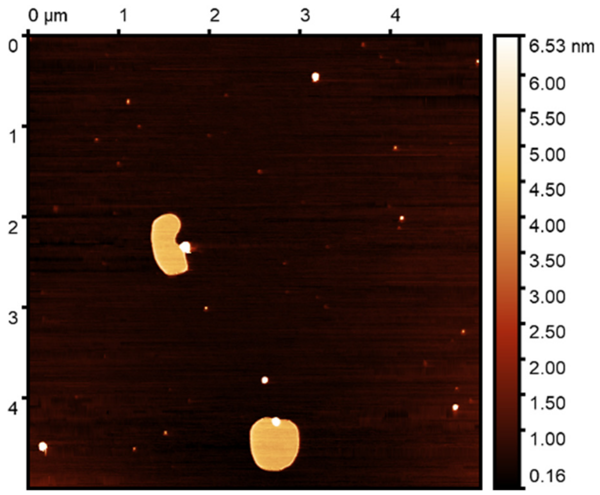

Figure S3. AFM images (topography) of *r*-BTPT obtained by evaporation of 10 μl solution at mica substrate.

2. In vitro ROS generation and accumulation of NPs

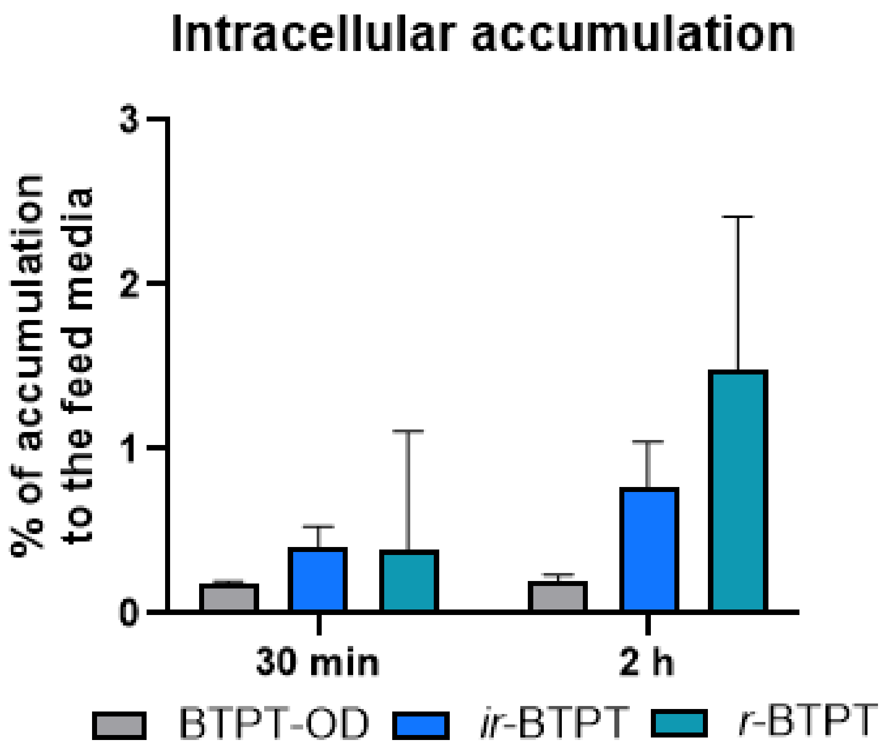

Figure S4. Intracellular accumulation of BTPT-OD, *r*-BTPT and *ir*-BTPT NPs in human breast adenocarcinoma Sk-Br-3, 10 µM and 0.5 µM for BTPT-OD and *r*-BTPT and *ir*-BTPT NPs, respectively, 30 min and 2 h incubation. The accumulation was measured in cell lysate in DMSO, ex. 670 nm, em. 850 nm. The cell lysate data were normalized to the fluorescence of the initial suspensions in DMSO; data are the mean ± SEM.

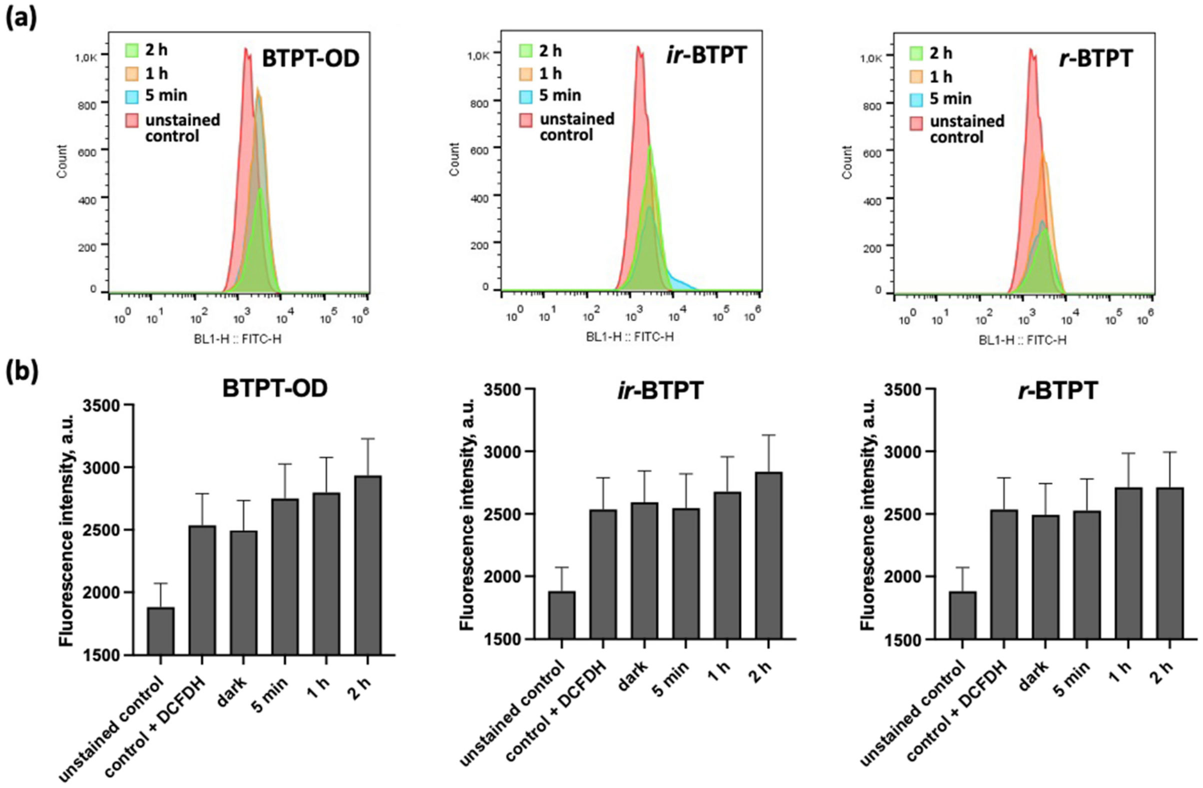

Figure S5. ROS generation of BTPT-OD, *r*-BTPT and *ir*-BTPT NPs in human breast carcinoma Sk-Br-3 cells evaluated by DCFDH staining. The concentration was 8 μM for BTPT-OD and 0.4 μM for *r*-BTPT and *ir*-BTPT NPs (near IC_{50} value), 5 min, 1 h and 2 h incubation. Cells were irradiated with 730 nm for a total 30 J/cm^2 light dose. Primary flow cytometry curves for 10 μg/ml (a) and mean fluorescent signal for all concentrations (b). Flow cytometry data, 20,000 events in each sample, data are the mean ± SD.

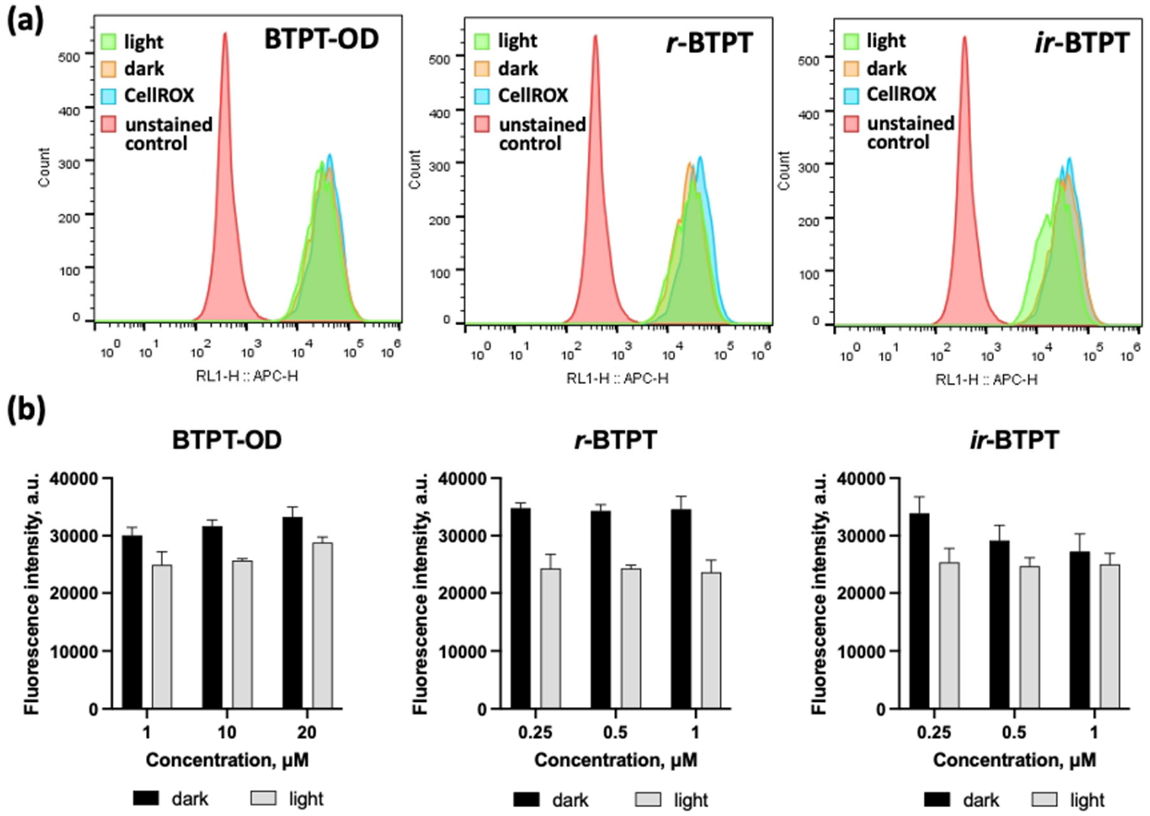

Figure S6. ROS generation of BTPT-OD, *r*-BTPT and *ir*-BTPT NPs in human breast carcinoma Sk-Br-3 cells evaluated by CellROX™ Deep Red staining. The concentration was 1 µM and 0.25 µM (sub-IC_{50} value), 10 µM and 0.5 µM (near IC_{50} value), and 20 µM for BTPT-OD and 1 µM for *r*-BTPT and *ir*-BTPT NPs (over IC_{50} value), respectively, 2 h incubation. Cells were irradiated with 730 nm for a total 30 J/cm^2 light dose. Primary flow cytometry curves for 10 µg/ml (a) and mean fluorescent signal for all concentrations (b). Flow cytometry data, 10,000 events in each sample, data are the mean ± SD.
